# Synthetic Cannabinoids and Its Association With Persistent Negative Symptoms of Schizophrenia

**DOI:** 10.7759/cureus.10329

**Published:** 2020-09-09

**Authors:** Ritvij Satodiya, Nikhil Palekar

**Affiliations:** 1 Child and Adolescent Psychiatry, New York University Grossman School of Medicine, New York, USA; 2 Psychiatry, Stony Brook University Hospital, Stony Brook, USA

**Keywords:** synthetic cannabinoids, schizophrenia and other psychotic disorders, negative symptoms, k2, spice

## Abstract

Schizophrenia has a multidomain symptom cluster, including positive, negative, and cognitive symptoms. Synthetic cannabinoids (SC) commonly perpetuate the positive symptoms of schizophrenia. We present a case of predominant negative symptoms following the use of SC even though our patient had a consistent history of experiencing positive symptoms in the past. The hypoactive dopaminergic system in the prefrontal cortex can induce negative symptoms in schizophrenia. However, the modulating properties of SC on cannabinoid receptors can feed into the negative symptom progression. The psychoactive properties of SC need further research to understand its clinical characteristics.

## Introduction

The current conceptualization of schizophrenia includes three major symptom domains: positive, negative, and cognitive. Although early schizophrenia studies have focused on positive symptoms, negative symptoms lend to poorer prognosis, increased psychotic episodes, and greater functional impairment [1]. Recently, the use of synthetic cannabinoids (SC), commonly known as “K2” or “Spice,” is becoming increasingly popular. With growing popularity, there has been much interest in its impact on psychotic symptom exacerbation [2]. Like its cannabinoid counterpart, SC also appears to perpetuate positive symptoms of schizophrenia [3]. While delta-tetrahydrocannabinol component of cannabis is a partial agonist, SC is a full agonist at cannabinoid receptors with a higher affinity towards CB1 receptors [4]. The limited evidence of SC on negative symptoms captivated the interest in this case of schizophrenia who developed significant negative symptoms with chronic use of SC.

## Case presentation

Our patient is a 32-year-old, single, domiciled, unemployed, Caucasian male with a history of schizophrenia and cannabis use disorder, who was admitted to the inpatient unit for deteriorating quality of life and notable k2 (three to four times per week) use. He did not exhibit any signs of delusions, hallucinations, paranoia, disorganized behavior, or disorganized speech during admission. However, he showed prominent signs of monotone speech, minimal gestures, social withdrawal, lack of spontaneity, blunted affect, and avolition. He did not show signs of catatonia including mutism, immobility, stupor, negativism, or posturing. He denied symptoms consistent with depression or mania and denied suicidal or homicidal ideations. He started smoking k2 (three to four times per week) approximately a year prior to this admission with no concurrent cannabis use except prior use. He denied using alcohol or any other drugs. He had no acute or chronic medical conditions and denied any allergies.

His first psychiatric manifestation was at age 18 years, which required hospitalization for paranoia and disruptive behavior. He was diagnosed with schizophrenia with six psychiatric hospitalizations for worsening paranoia and safety concerns in the community. Most strikingly, he experienced predominantly positive symptoms of schizophrenia prior to k2 use. However, he became more withdrawn, isolative, and lacked interest in social activities and eventually was unable to maintain basic activities of daily living following k2 use. Despite his presentation may resemble with catatonia, there were no signs of immobility, stupor, staring, grimacing, echopraxia, stereotypy, mannerism, rigidity, negativism, waxy flexibility, or excitement. 

## Discussion

Our patient liked k2 which is a man-made functional analog of cannabis that binds to cannabinoid receptors type 1 (cbr1) with much higher affinity and potency than delta-9-tetrahydrocannabinol. They also lack protective effects of cannabidiol including anxiolytic and antipsychotic effects [5,6].

Our patient has a history of chronic cannabis use with the development of schizophrenia, which is consistent with the evidence of elevated risk of psychosis with cannabis [6]. However, the gradual emergence of negative symptoms within the first month after daily k2 use highlights a significant change in his conventional symptomatology. Catatonia was ruled out given the similarity of the presentation. His negative symptoms are unlikely to chronic cannabis use considering his experience of primarily positive symptoms in past and complete discontinuation of cannabis use. The development of negative symptoms is plausible from the natural progression of schizophrenia. However, this seems unlikely given the temporal relationship between the development of negative symptoms with the onset of SC use.

These negative symptoms are typically attributed to the hypoactive dopaminergic system, particularly in the prefrontal cortex. Also, they persisted longer than usual symptom-course of natural cannabis [7,8]. Chronic use of SC can cause adaptive changes in the cannabinoid receptor-mediated signaling cascade that can result in more adverse effects that are not much studied yet [9].

There is large heterogeneity in SC. The precise chemical make-up of his compound is unknown as there are at least 200 different SC that have been isolated and currently being studied for their psychoactive properties [6].

## Conclusions

SC abuse is an emerging problem given its low cost and easy accessibility. One of the reason may be its higher potency to produce psychotogenic effects in chronic cannabis user who have developed tolerance to cannabis. Although cannabis and its cognate chemicals are associated with the development of psychosis with likely positive symptoms, we share our findings of predominant presentations with negative symptoms on SC use. Further research is required to study the biological composition of SC and its interactions with the endocannabinoid system to understand the clinical characteristics.
